# Novel compound heterozygous *POR* variants in a neonate with Antley-Bixler syndrome and 46,XY DSD: a case report and literature review

**DOI:** 10.3389/fendo.2026.1896956

**Published:** 2026-08-28

**Authors:** Wenting Zhang, Junfeng Zeng, Yanjin Li, Cailian Xie, Zhihao Sun, Guizhen Lyu, Tizhen Yan, Xiaoling Huang

**Affiliations:** 1 Neonatal Disease Screening Center, Dongguan Maternal and Child Health Care Hospital, Dongguan, Guangdong, China; 2 Dongguan Key Laboratory of Screening, Diagnosis and Treatment of Neonatal Genetic and Metabolic Diseases, Dongguan, Guangdong, China; 3 Central Laboratory, Dongguan Maternal and Child Health Care Hospital, Dongguan, China; 4 Dongguan Molecular Diagnostic Technology and Infectious Disease Medical Test Engineering Research Center, Dongguan Labway Clinical Laboratory Co., Ltd., Dongguan, Guangdong, China; 5 Institute of Reproduction and Genetics, Dongguan Maternal and Child Health Care Hospital, Dongguan, Guangdong, China; 6 Dongguan Key Laboratory of Reproduction and Birth Defects Prevention and Control, Dongguan Maternal and Child Health Care Hospital, Dongguan, Guangdong, China

**Keywords:** Antley-Bixler syndrome, congenital adrenal hyperplasia, cytochrome P450 reductase deficiency, disorder of sex development, *POR* gene

## Abstract

**Background:**

Cytochrome P450 oxidoreductase deficiency (PORD) is an ultra-rare autosomal recessive disorder within the congenital adrenal hyperplasia (CAH) spectrum, characterized by a broad clinical spectrum involving steroidogenesis defects, genital anomalies, and skeletal abnormalities.

**Case presentation:**

We report a phenotypically female neonate with a 46,XY karyotype whose postnatal diagnostic evaluation was initiated after newborn screening revealed elevated 17-hydroxyprogesterone (17-OHP) concentration. The patient presented with mild hypertelorism, mild nasal hypoplasia, and low-set bilateral ears, along with female external genitalia consistent with disorder of sex development (DSD) and anal atresia. Radiological evaluation revealed femoral bowing and subsequent fracture. The craniofacial and skeletal abnormalities were consistent with the features of Antley-Bixler syndrome (ABS). Endocrine evaluation revealed elevated progesterone, markedly reduced testosterone, and secondary hyperaldosteronism. Genetic analysis identified three novel variants in the *POR* gene (NM_001395413.1): the patient harbored a paternal c.1187_1195dup (p.Pro396_Glu398dup) variant and two maternally inherited variants in *cis*, c.1447G>A (p.Gly483Ser) and c.1806 + 4_1806 + 28del. Protein structural modeling predicted that the p.Pro396_Glu398dup and p.Gly483Ser may disrupt the flavin adenine dinucleotide (FAD)–binding domain. RNA sequencing (RNA-seq) confirmed that the intronic variant c.1806 + 4_1806 + 28del caused aberrant splicing, resulting in partial intron retention and predicted impairment of the nicotinamide adenine dinucleotide phosphate (NADPH)–binding domain. According to American College of Medical Genetics and Genomics (ACMG) guidelines and incorporating functional evidence, c.1187_1195dup and c.1806 + 4_1806 + 28del were reclassified as likely pathogenic (LP), whereas c.1447G>A remained a variant of uncertain significance (VUS).

**Conclusions:**

This study describes a neonate with PORD caused by three novel POR variants and expands the known clinical spectrum of PORD by identifying rare manifestations including anal atresia and hearing loss. RNA-seq provided valuable functional evidence for variant interpretation and facilitated accurate molecular diagnosis. These findings highlight the importance of integrating genetic phasing, transcript-level functional analysis, and comprehensive clinical evaluation for precise diagnosis and counseling in rare endocrine disorders.

## 1 Introduction

Congenital adrenal hyperplasia (CAH) is primarily caused by mutations in genes encoding steroidogenic enzymes or their auxiliary factors. Cytochrome P450 oxidoreductase (POR) is a flavoprotein that donates electrons to all microsomal cytochrome P450 enzymes, including key steroidogenic enzymes CYP21A2 and CYP17A1 (1, 2). POR deficiency (PORD) is an ultra-rare autosomal recessive disorder belonging to the CAH spectrum, with fewer than 200 reported genetically verified cases worldwide (3, 4). Due to impaired electron transfer to multiple cytochrome P450 enzymes, PORD exhibits substantial phenotypic variability, ranging from isolated steroidogenic abnormalities to complex developmental disorders involving the endocrine system, genital development, craniofacial structures, and the skeleton. (e.g., femoral bowing, radio-ulnar synostosis) (5, 6). When accompanied by characteristic craniofacial and skeletal abnormalities, including femoral bowing and radio-ulnar synostosis, the condition is referred to as Antley-Bixler syndrome (ABS) (7–9). The diagnosis of PORD remains challenging because of its phenotypic heterogeneity and the frequent identification of variants of uncertain significance (VUS), particularly in prenatal genetic testing. Functional assays capable of directly evaluating the biological consequences of genetic variants are therefore essential for accurate molecular classification.

Here, we provide a detailed report of a neonate with PORD associated with ABS and 46,XY DSD, carrying three novel POR variants. Through integrated genetic analysis, protein structural modeling, and RNA sequencing (RNA-seq), we investigated the potential pathogenic mechanisms of these variants. The aim of this study was to comprehensively characterize the clinical, hormonal, genetic, and functional features of this patient and to compare the findings with previously reported genetically confirmed PORD cases, thereby expanding the understanding of the phenotypic spectrum of PORD and providing insights into variant interpretation, diagnosis, and genetic counseling.

## 2 Case report

### 2.1 Clinical presentation

The patient was assigned a female gender at birth and was delivered at 39 weeks and 3 days of gestation following oxytocin-induced labor. Birth weight was 2.57 kg, and birth length was 50 cm. Prenatal ultrasonography at 23 weeks of gestation revealed angulation of the left femur. Postnatally, the patient underwent uncomplicated surgical repair for anal atresia. Newborn screening via dried blood spot showed elevated 17-hydroxyprogesterone (17-OHP) concentration of 36.9 nmol/L (reference value <13.9 nmol/L), prompting further endocrine and genetic evaluation. The parents are healthy and non-consanguineous. The mother has a history of one unexplained early miscarriage but reported no signs of virilization during pregnancy. The patient’s two older sisters have normal growth and development. Physical examination revealed mild hypertelorism, a mildly depressed nasal bridge, and bilateral low-set ears. The anterior fontanelle was soft and flat. Cardiopulmonary and abdominal examinations were unremarkable. External genitalia exhibited typical female morphology without marked hyperpigmentation.

### 2.2 Endocrine and biochemical evaluation

Comprehensive endocrine and biochemical evaluations were performed (**Table 1**). Assessment of adrenal function revealed evidence of impaired steroidogenesis. Supine aldosterone (ALDO) levels were markedly elevated (1910.70 pg/mL at 2 months and 578.89 pg/mL at 4 months; reference range, 10.00–160.00 pg/mL), accompanied by increased plasma renin activity (PRA: 3.91 ng/mL/h at 4 months; reference range, 0.15–2.33 ng/mL/h), suggesting secondary hyperaldosteronism.

**Table 1:** Endocrine and biochemical evaluation, treatment, and developmental profile.

Items		7–10 d	1 m	2 m	3 m	4 m	9 m	14 m	Reference range (male)
ALDO and PRA (Regular diet, supine)	ALDO (pg/mL)	–	–	1910.70 ↑	–	578.89 ↑	–	–	Supine: 10.00–160.00 Upright: 40.00–310.00
	Angiotensin I (37°C) (ng/mL)	–	–	–	–	4.62	–	–	
	Angiotensin I (4°C) (ng/mL)	–	–	–	–	0.71	–	–	
	PRA (ng/mL/h)	–	–	–	–	3.91 ↑	–	–	Regular diet, upright: 1.31–3.95 Regular diet, supine: 0.15–2.33
ACTH and cortisol	ACTH (pg/mL)	25.19	–	89.95 ↑	–	–	–	–	7.20–63.40
	COR (μg/dL)	AM: 0.96 ↓	PM: 3.81	PM: 6.49	AM: 0.21 ↓	AM: 7.86	AM: 9.64	AM: 5.81	AM: 4.26–24.85 PM: 2.90–17.30
Sex hormones	17-OHP (nmol/L)	10.2	14.2 ↑	10.2	–	0.7	4.2	11.1	< 13.9
	eE2 (pg/mL)	22.34	–	–	–	–	–	–	0–39.8
	FSH (mIU/mL)	5.80	–	–	–	–	–	–	Newborn: < 1–2.4 2w-1y: < 1-20
	PRGE (ng/mL)	18.13 ↑	–	–	–	–	–	–	0.28–1.22
	T (ng/dL)	< 7.00 ↓	8.77 ↓	–	–	–	–	–	123.06–813.86
	LH2 (mIU/mL)	0.88	–	–	–	–	–	–	0–20y: 0–6.0
	PRL (ng/mL)	60.47 ↑	–	–	–	–	–	–	2.1–17.7
	A4 (nmol/L)	< 0.44	< 0.44	< 0.44	–	–	–	–	0–1m: ≤ 10.12 1–12m: ≤ 2.72 1y–5y: ≤ 1.78
	DHEA-S (μg/dL)	454.27	290.55	66.23	–	–	–	–	0–1w: 107.00–607.55 1–4w: 31.20–431.44 1–12m: 3.20–124.56
	AMH (ng/mL)	52.80	–	–	–	–	–	–	< 11y: 38.79–294.19
	INH B (pg/mL)^a^	215.40	–	–	–	–	–	–	5–10y: 21–166 10–14y: 41–328 14–18y: 135–368 18–80y: 15–295
Serum ions	K^+^ (mmol/L)	5.15	–	–	4.87	–	4.60	–	3.5–5.3
	Na^+^ (mmol/L)	140.0	–	–	137.0	–	135.9 ↓	–	137–147
	Cl^−^ (mmol/L)	99.2	–	–	105.2	–	101.1	–	96.0–108.0
	Ca^2+^ (mmol/L)	1.93 ↓	–	–	2.26	–	–	–	2.25–2.67
	Mg^2+^ (mmol/L)	1.08 ↑	–	–	0.93	–	–	–	0.80–1.00
	Pi (mmol/L)	2.36 ↑	–	–	1.76	–	–	–	1.45–2.10
Treatment		–	–	–	Started at 3 months old: Medication: Hydrocortisone (5 mg/tablet), total daily oral dose 2.5 mg split into three portions (1/4, 1/8, 1/8 tablet)				–
Developmental profile		–	–	–	Height 62cm	5 months: height 65.2 cm, weight 7.4 kg; 6 months: able to sit independently; 12 months: 73 cm, 9 kg, stands steadily		Walks with support, DQ > 100 ^b^	–

PRA = Angiotensin I (37°C) − Angiotensin I (4°C); ALDO, aldosterone; PRA, plasma renin activity; ACTH, adrenocorticotropic hormone; COR, cortisol; 17-OHP, 17α-hydroxy progesterone; eE2, E2 estradiol; FSH, follicle-stimulating hormone; PRGE, progesterone; T, testosterone; LH2, luteinizing hormone; PRL, prolactin; A4, 4-androstenedione; DHEA-S, dehydroepiandrosterone sulfate; AMH, anti-Müllerian hormone; INH B, inhibin B; Age of the patient: d, days; m, months; y, years; K^+^, potassium; Na^+^, sodium; Cl^-^, chloride; Ca^2+^, serum total calcium; Mg^2+^, magnesium ion; Pi, inorganic phosphorus;-, not tested, untreated or not recorded;

^a^ Local reference ranges for INH B in males younger than 5 years have not been established in our laboratory;

^b^ DQ, developmental quotient, was assessed using Developmental Scale for Children Aged 0–6 Years (National health standard WS/T 580-2017, Er-Xin Scale II).

At 7 days of age, adrenocorticotropic hormone (ACTH) levels were within the reference range; however, ACTH increased to 89.95 pg/mL at 2 months (reference range, 7.20–63.40 pg/mL). Serial measurements of serum cortisol levels revealed considerable variability, with decreased concentrations observed at 7 days (0.96 μg/dL) and 3 months (0.21 μg/dL) (reference ranges: 4.26–24.85 μg/dL in the morning and 2.90–17.30 μg/dL in the afternoon).

The sex hormone profile showed abnormalities consistent with impaired steroid biosynthesis. Serum 17-OHP was within the reference range at 7 days of age but increased to 14.2 nmol/L at 1 month (reference value, <13.9 nmol/L). Progesterone was elevated at 7 days of age (18.13 ng/mL; reference range, 0.28–1.22 ng/mL). Testosterone levels were markedly decreased, measuring <7 ng/dL at 7 days and 8.77 ng/dL at 1 month (reference range, 123.06–813.86 ng/dL). In contrast, prolactin (PRL) was elevated at 7 days of age (60.47 ng/mL; reference range, 2.1–17.7 ng/mL). Estradiol, follicle-stimulating hormone (FSH), luteinizing hormone (LH), 4-androstenedione (A4), dehydroepiandrosterone sulfate (DHEA-S), and anti-Müllerian hormone (AMH) levels were within the reference ranges.

Serum electrolyte analysis revealed no significant abnormalities. Potassium, sodium, and chloride levels remained within normal ranges during follow-up, except for a mildly decreased sodium (Na^+^) concentration at 9 months of age (135.9 mmol/L; reference range, 137–147 mmol/L).

### 2.3 Imaging examinations

**Figure 1:**
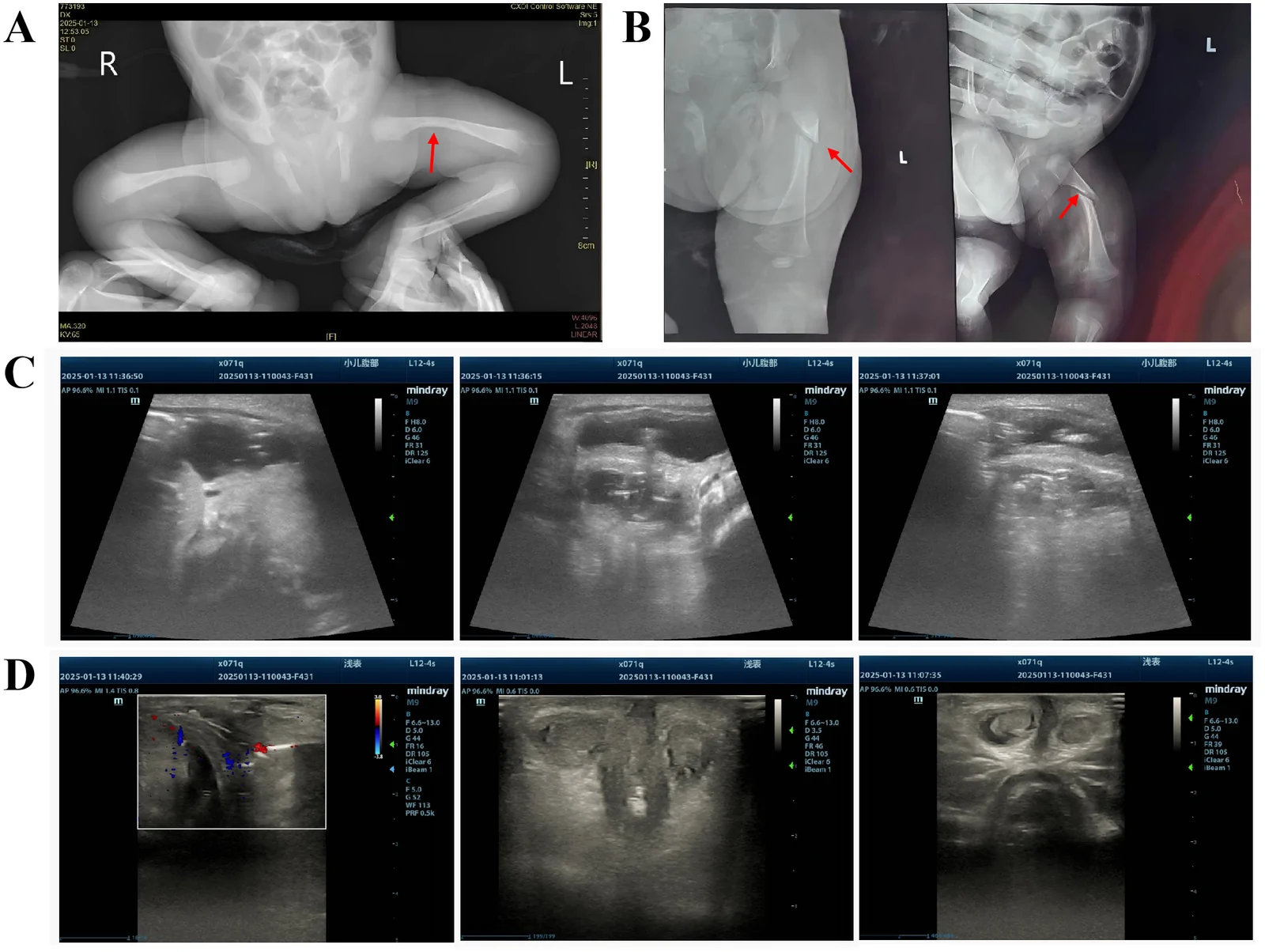
Imaging of the patient’s skeletal system, pelvis, and perineum. **(A)** Radiograph on day 1 after birth shows curvature of the left femur (arrow). **(B)** Radiograph on day 42 after birth shows a fracture of the left femur (arrow). Color Doppler ultrasound reveals no clear imaging of the uterus and ovaries **(C)**, and a testis-like echo in the perineal region **(D)**.

Radiography performed on postnatal day 1 confirmed left femoral bowing (**Figure 1A**), while a fracture of the left femur was identified on postnatal day 42 (**Figure 1B**). Pelvic ultrasonography demonstrated no detectable uterus or ovaries (**Figure 1C**), although a testis-like structure was visualized within the perineum (**Figure 1D**). Echocardiography revealed a patent foramen ovale and moderate tricuspid regurgitation. Renal ultrasonography showed mild separation of the right renal pelvis.

### 2.4 Hearing assessment

The patient failed neonatal hearing screening in both ears. A diagnostic assessment at 3 months of age revealed bilateral mild conductive hearing loss, with auditory brainstem response (ABR) thresholds of 35 dB nHL on the left and 40 dB nHL on the right and flat 1000 Hz tympanometry curves bilaterally.

### 2.5 Genetic analysis and pathogenicity assessment

#### 2.5.1 Chromosomal analysis and molecular genetic testing

Retrospective review of raw amniotic fluid karyotype data revealed an underlying karyotype of 46,XY. Consistent with institutional guidelines prohibiting disclosure of fetal sex in prenatal clinical reports, the formal prenatal report was documented as 46,XN. Postnatal peripheral blood karyotyping confirmed the 46,XY karyotype. The *SRY* gene was positive without detected pathogenic variants.

#### 2.5.2 Variant identification and segregation analysis

**Figure 2:**
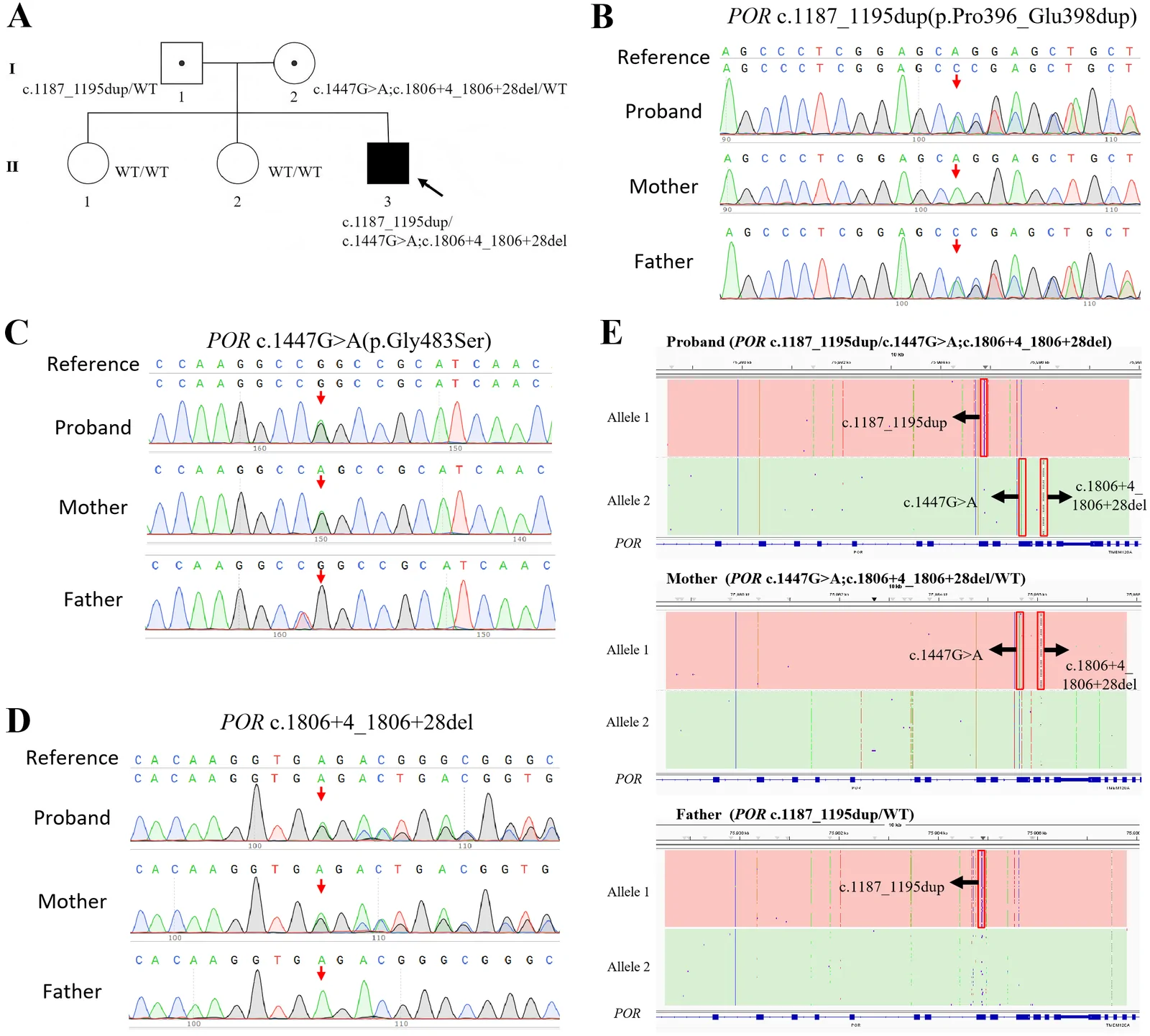
Pedigree, Sanger sequencing and Long-read sequencing validation results. **(A)** Pedigree of the family. The proband (II-3) is a compound heterozygote. The father (I-1) and mother (I-2) are carriers of different variants, respectively. The two older sisters (II-1, II-2) have a normal genotype. WT, wild type. (**B–D**) Sequencing chromatograms showing the corresponding variant sites in the proband and parents. **(B)** The heterozygous c.1187_1195dup (p.Pro396_Glu398dup) variant inherited from the father. **(C)** The heterozygous c.1447G>A (p.Gly483Ser) variant inherited from the mother. **(D)** The heterozygous c.1806 + 4_1806 + 28del variant inherited from the mother. The Sanger sequencing results for the two older sisters are not shown here. **(E)** Long-read sequencing results confirmed the allelic configuration and *cis* linkage of POR gene variants.

Whole exome sequencing and Sanger sequencing validation revealed that the patient carries three heterozygous variants in the *POR* gene (NM_001395413.1) (**Table 2**). Family segregation analysis confirmed an autosomal recessive inheritance pattern (**Figure 2A**) with a specific allelic configuration: the c.1187_1195dup (p.Pro396_Glu398dup) variant was inherited from the father. Long-read sequencing verification confirmed that the two other variants, c.1447G>A (p.Gly483Ser) and c.1806 + 4_1806 + 28del, were located on the same maternal allele (in *cis*) (**Figures 2B–E**). Therefore, the patient was confirmed to be a compound heterozygote carrying one paternal mutant allele and one maternal allele harboring two variants.

**Table 2:** Evaluation and reclassification of POR gene variants according to ACMG guidelines.

Gene	Variants	Gene subregion	Frequency	Origin	Pre-upgrading		Post-upgrading		
					Pathogenicity	Evidence		Pathogenicity	Evidence
*POR*	c.1187_1195dup (p.Pro396_Glu398dup)	Exon 11	0.00000179969	Father	VUS	PM2_Supporting+ PM4+PP4		LP	PM2_Supporting+ PM3_Moderate+PM4+PP4
*POR*	c.1447G>A (p.Gly483Ser)	Exon 13	0.00019253	Mother	VUS	PM2_Supporting+ PP3+PP4		VUS	PM2_Supporting+ PP3+PP4
*POR*	c.1806 + 4_1806 + 28del	Intron 14	–	Mother	VUS	PM2_Supporting+PP4		LP	PVS1+PM2_Supporting+ PP4

Genome Reference Version: hg38/GRCh38; Transcript: NM_001395413.1; VUS, variant of uncertain significance; LP, likely pathogenic.

Detailed descriptions of ACMG evidence criteria are provided in the main text.

#### 2.5.3 Functional prediction and pathogenicity assessment

**Figure 3:**
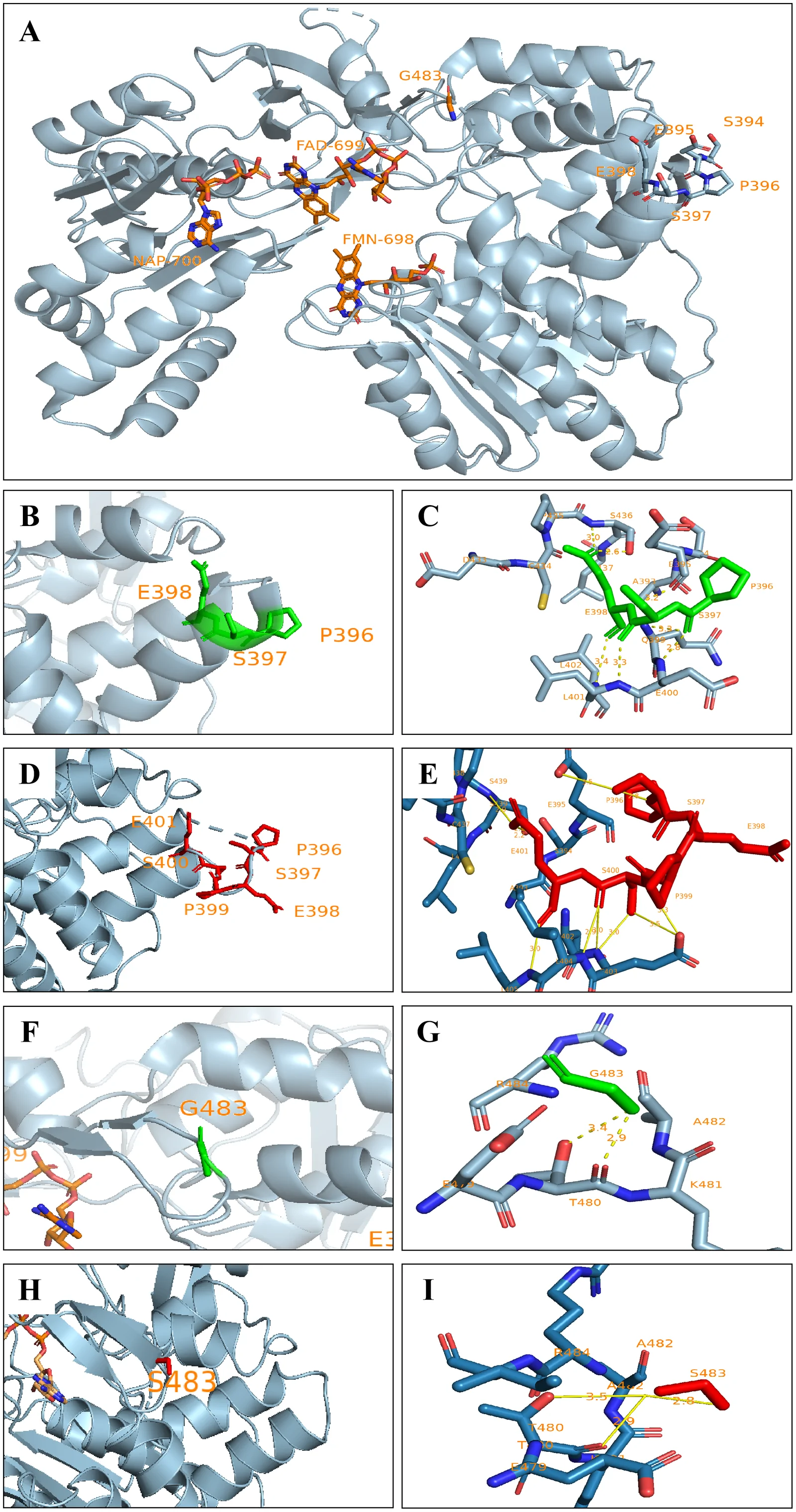
Local structural models of the wild-type and mutant POR protein. **(A)** Wild-type POR protein crystal structure (PDB ID: 5FA6). Amino acid residues G483 and P396-E398, as well as three ligands, are annotated in the figure. **(B)** Wild-type Pro396_Glu398 (green) forms an alpha-helical structure. **(C)** Interactions of p.Pro396_Glu398 (green) with surrounding residues. **(D)** p.Pro396_Glu398dup mutant (red), with the duplication predicted to convert the local alpha-helical structure into a random coil. **(E)** Interactions of the p.Pro396_Glu398dup mutant (red) with surrounding residues. **(F)** Wild-type Gly483 (green) forms a random coil structure with adjacent amino acids. **(G)** Interactions of Gly483 (green) with surrounding residues. **(H)** p.Gly483Ser mutant (red), where the conserved glycine is replaced by serine, predicted to convert the local structure from a random coil to a beta-sheet. **(I)** Interactions of the p.Gly483Ser mutant (red) with surrounding residues. Yellow dashed lines and yellow solid lines indicate hydrogen bonds formed between the wild-type and mutant residues, respectively, with residues within 5 Å.

1. Protein structure analysis (**Figure 3**): Protein structure prediction was performed using AlphaFold 3 (DeepMind) (10), accessed via the EMBL-EBI AlphaFold3 web server (https://alphafoldserver.com/). AlphaFold 3 automatically retrieved experimental structures deposited in the Protein Data Bank (PDB) as structural templates. Primary structural confidence metrics, predicted local distance difference test (pLDDT) and predicted aligned error (PAE) generated intrinsically by AlphaFold 3, were extracted for model quality assessment. The predicted template modeling (pTM) score and the interface predicted template modeling (ipTM) score are applied to measuring the accuracy of the entire structure. The top-ranked Model 0 was selected for analysis. Protein structure visualization and labeling of molecular interactions were performed using PyMOL (Version 2.5.0).

In this study, the model for POR c.1187_1195dup (p.Pro396_Glu398dup) yielded an iPTM of 0.98 and a pTM of 0.81; the low fraction of disordered residues (0.09) and absence of steric clashes support the high reliability of this predicted structure. Similarly, the structural prediction model of POR c.1447G>A (p.Gly483Ser) achieved an overall iPTM of 0.98 and a pTM of 0.81, with only 10% disordered residues and no steric clashes observed, indicating high global structural confidence.

The crystal structure of wild-type POR (PDB ID: 5FA6) was used as a structural reference (**Figure 3A**). Homology modeling predicted that p.Pro396_Glu398dup, located within the FAD-binding domain, may disrupt a local alpha-helical and induce conversion to a random coil configuration (**Figures 3B–E**). The p.Gly483Ser variant, located adjacent to the FAD-binding domain within a highly conserved region, was predicted to alter the local secondary structure from a random coil to a beta-sheet, potentially affecting cofactor binding (**Figures 3F–I**).

2. Splicing analysis: To predict the potential splicing effect of the variant, we first performed *in-silico* splicing prediction using the SpliceAI algorithm. Subsequently, transcript-level verification was conducted via RNA-seq. Total RNA was extracted from peripheral blood samples using the PAXgene Blood RNA Kit (QIAGEN, Hilden, Germany). mRNA libraries were constructed with the Hieff NGS® Ultima Dual-mode mRNA Library Prep Kit (Yeasen, Shanghai, China) and circularized using the VAHTS Circularization Kit (Vazyme, Nanjing, China), followed by high-throughput sequencing on the DNBSEQ-T7RS platform (MGI, Shenzhen, China) with paired-end 150 bp reads, generating 103.54 million raw reads (15.53 G bases). Raw sequencing data were quality-controlled and trimmed using fastp to obtain high-quality clean reads. Clean reads were aligned to the hg19 (GRCh37) reference genome using STAR (v2.5.3a), and differential alternative splicing events were further analyzed via the rMATS pipeline.

**Figure 4:**
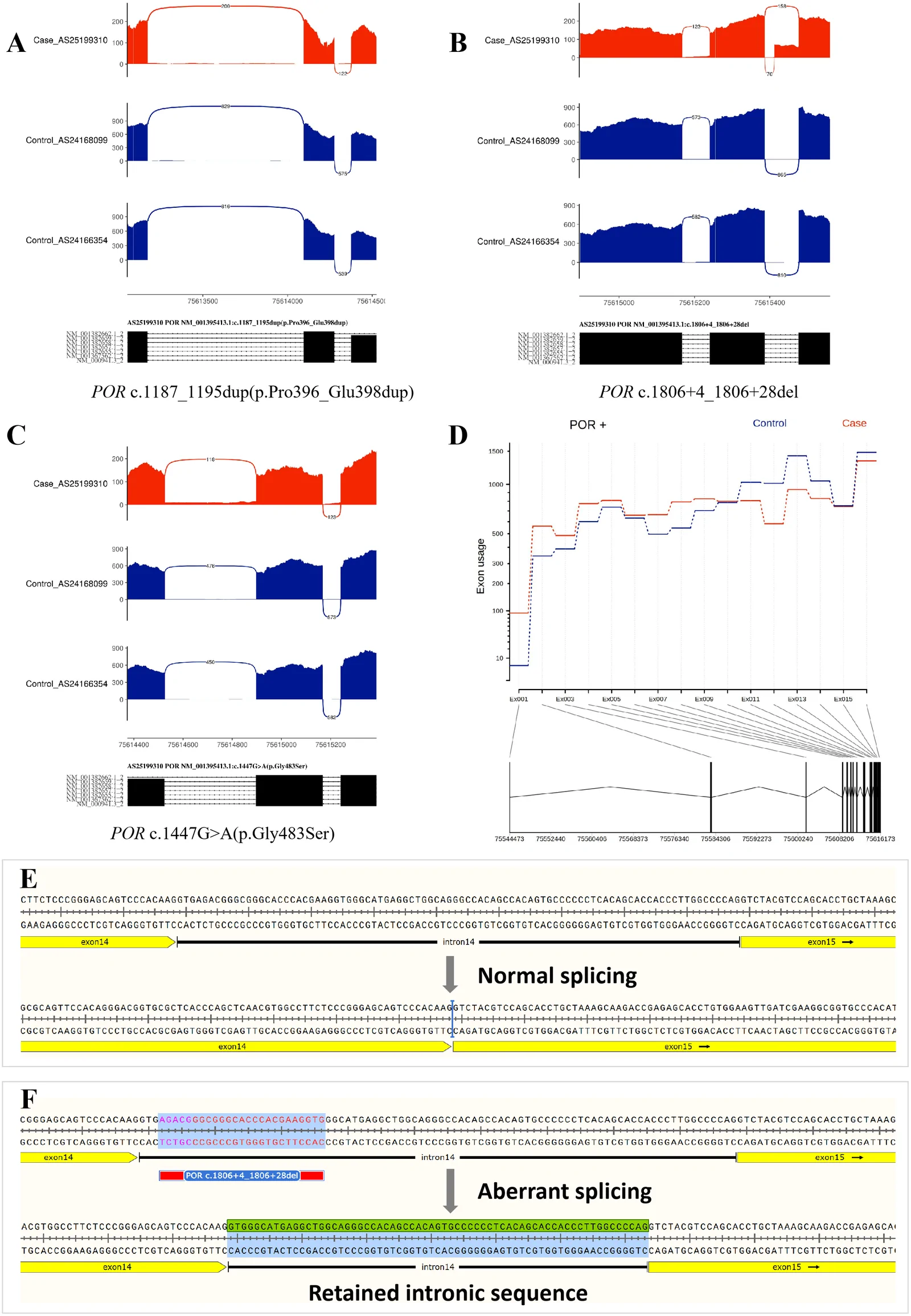
Results of alternative splicing analysis for the three variants (SpliceAI) and RNA-seq results confirming partial intronic retention for the c.1806 + 4_1806 + 28del variant. **(A)** No aberrant alternative splicing event was detected for the *POR* gene c.1187_1195dup (p.Pro396_Glu398dup) variant in the patient. SpliceAI showed no Δscore prediction for this site. **(B)** An aberrant alternative splicing event was detected for the *POR* gene c.1806 + 4_1806 + 28del variant in the patient. SpliceAI predicted an Acceptor Loss with a Δscore of 0.48 at position 76 bp, a Donor Loss with a Δscore of 1.00 at position 3 bp, and a Donor Gain with a Δscore of 0.88 at position 28 bp. **(C)** No aberrant alternative splicing event was detected for the *POR* gene c.1447G>A (p.Gly483Ser) variant in the patient. SpliceAI showed no Δscore prediction for this site. **(D)** RNA-seq results showed partial intronic retention of intron 14 (65 bp, near the exon 15 boundary). Retained intronic sequence: GTGGGCATGAGGCTGGCAGGGCCACAGCCACAGTGCCCCCCTCACAGCACCACCCTTGGCCCCAG (chr7:75615412- 75615476). Schematic diagram illustrating normal splicing of intron 14 of the *POR* gene **(E)** and aberrant splicing and base retention induced by the c.1806 + 4_1806 + 28del variant **(F)**.

Integrated *in-silico* prediction and experimental RNA-seq analysis confirmed that the intronic variant c.1806 + 4_1806 + 28del results in aberrant splicing (**Figures 4A–C**), resulting in partial retention of 65 bp of intron 14 (**Figure 4D**), as shown in a schematic illustrating normal versus aberrant splicing (**Figures 4E, F**). This aberrant transcript is predicted to generate a truncated protein or lead to nonsense-mediated mRNA decay, thereby impairing the NADPH-binding domain of POR.

#### 2.5.4 ACMG classification and variant interpretation

Variant classification was performed according to the American College of Medical Genetics and Genomics (ACMG) standards and guidelines (11), alongside recommendations released by the ClinGen Sequence Variant Interpretation (SVI) working group (12–14). We incorporated functional evidence from RNA-seq and genetic phasing data demonstrating variants in *trans* to revise the initial variant classifications (**Table 2**). The ACMG evidence codes and corresponding justifications for each variant are listed as follows:

1. c.1806 + 4_1806 + 28del: Reclassified from VUS to Likely Pathogenic

ACMG evidences: PVS1 + PM2_Supporting + PP4

PVS1: The variant affects a splice region and was demonstrated by RNA-seq to cause abnormal splicing with partial retention of intron 14, supporting loss-of-function as the disease mechanism of POR.

PM2_Supporting: This variant has not been reported in relevant clinical cases and is not documented in gnomAD v2.1.1 and has not been reported in affected individuals.

PP4: The patient’s clinical features, including 46,XY DSD, skeletal abnormalities, and abnormal steroid profiles, are highly consistent with PORD.

2. c.1187_1195dup (p.Pro396_Glu398dup): Reclassified from VUS to Likely Pathogenic

ACMG evidences: PM2_Supporting + PM3_Moderate + PM4 + PP4

PM2_Supporting: This variant is absent from the Chinese population-specific genome database, ExAC, and 1000 Genomes Project databases. Its allele frequency in gnomAD is extremely low (0.00000179969).

PM3_Moderate: The variant was confirmed to be in trans with another likely pathogenic POR variant (c.1806 + 4_1806 + 28del) in an affected individual with an autosomal recessive disorder. Based on the ClinGen SVI point-based framework, the verified trans configuration provided moderate evidence.

PM4: The duplication alters the protein length without disrupting the open reading frame, suggesting a potential effect on protein structure.

PP4: The patient’s phenotype is highly consistent with PORD.

3. c.1447G>A (p.Gly483Ser): Remains classified as VUS

ACMG evidences: PM2_Supporting + PP3 + PP4

PM2_Supporting: The variant is absent from ExAC, 1000 Genomes Project, and Chinese population-specific databases, with a low allele frequency in gnomAD (0.00019253).

PP3: Multiple computational algorithms predict a deleterious effect on POR protein structure.

PP4: The patient’s phenotype is compatible with PORD.

### 2.6 Treatment and follow-up

The treatment course, follow-up outcomes, hormonal profiles, and growth parameters are summarized below (**Table 1**). The left femoral fracture was initially managed at a local hospital, and the patient subsequently achieved satisfactory clinical recovery.

Glucocorticoid replacement therapy was initiated at 3 months of age. The patient received oral hydrocortisone (5 mg/tablet) at a total daily dose of 2.5 mg. Following treatment initiation, adrenal hormone levels improved, with serum cortisol concentrations maintained within the reference range during follow-up.

Due to specific circumstances, formal audiological assessment has not yet been performed during follow-up. However, according to parental reports, the patient has demonstrated no obvious hearing impairment in daily life.

Regarding growth and development, the patient showed favorable progress during follow-up. At 3 months of age, the patient’s height was 62 cm. At 5 months, height and weight were 65.2 cm and 7.4 kg, respectively. The patient achieved independent sitting at 6 months of age. At 12 months, height and weight were 73 cm and 9 kg, respectively, and the patient was able to stand steadily. At 14 months, the patient was able to walk with support, and the developmental quotient (DQ) was >100. Overall, the patient demonstrated normal growth and age-appropriate developmental milestones during the follow-up period.

## 3 Discussion

PORD is an exceptionally rare disorder characterized by impaired electron transfer from NADPH to multiple microsomal cytochrome P450 enzymes involved in steroidogenesis and embryonic development. In this study, we describe a neonate with PORD presenting with ABS, 46,XY DSD, featuring characteristic craniofacial dysmorphism (mild hypertelorism, mildly depressed nasal bridge, bilateral low-set ears), skeletal abnormalities, and a complex steroid profile indicative of combined 21-hydroxylase and 17α-hydroxylase/17,20-lyase deficiencies. The case’s novelty lies in the identification of three POR variants in a compound heterozygous state (paternal duplication and a maternal allele harboring both a missense and a splicing variant), none of which have been previously reported. Through protein modeling and, critically, RNA-seq analysis, we provide solid transcriptional functional evidence supporting the pathogenicity of two reclassified variants, offering molecular insights into PORD pathogenesis.

The genetic findings in this study establish a clear genotype–phenotype correlation that accounts for the patient’s multi-system clinical manifestations. POR serves as an essential obligate electron donor for key steroidogenic enzymes CYP21A2 and CYP17A1 (15). Structurally, the paternal duplication (p.Pro396_Glu398dup) and the maternal missense variant (p.Gly483Ser) are both predicted to disrupt the FAD-binding domain responsible for NADPH electron acceptance (4, 16), while maternal splicing variant (c.1806 + 4_1806 + 28del) is predicted to impair the NADPH-binding domain. Biallelic damage to these core functional domains synergistically likely diminishes POR enzymatic activity, resulting in simultaneous impairment of multiple steroidogenic pathways and the typical endocrine and sexual developmental phenotypes observed in this patient. Notably, the p.Gly483Ser variant remains classified as VUS. Since it resides in *cis* with the severe splicing variant on the same maternal allele, its independent phenotypic contribution cannot be reliably distinguished; it may act as a mild modifier variant or be phenotypically masked by the dominant splicing defect, which further explains the complex and variable genotype-phenotype heterogeneity of PORD.

To further characterize the clinical spectrum of PORD, we reviewed genetically confirmed cases reported in the literature up to January 2026. Among 186 reported cases (**Supplementary Tables 1**, **S2**; summarized in **Table 3**), missense variants represented the predominant variant type (70.1%), with p.R457H being the most frequently reported recurrent variant (36.2%) (**Supplementary Figures 1**, **S2**). The most consistent clinical manifestations included hormonal abnormalities (93.1%), craniofacial and skeletal abnormalities (77.2%), and genital abnormalities (72.0%), confirming the characteristic phenotype of PORD. POR-dependent pathways participate not only in steroidogenesis but also in cholesterol biosynthesis and retinoic acid metabolism, which are important for embryonic skeletal development. Disruption of these pathways may contribute to abnormalities of osteogenesis and chondrogenesis, providing a biological rationale for craniofacial and skeletal manifestations in PORD (17, 18).

**Table 3:** Clinical phenotypes of patients with PORD.

	Number of cases	Frequency (%)
Total number of cases ^*^	186	–
Gender (46,XY/46,XX)	73/109 **^#^**	–
Age (fetus, infant to adolescence/adult)	142/44	–
External genital malformations	134 (*n* = 186)	72.04 (134/186)
Hormonal abnormalities or delayed puberty	162 (*n* = 174)	93.10 (162/174)
Craniofacial, skeletal, or limb anomalies	142 (*n* = 184)	77.17 (142/184)
Ovarian cysts ^†^	45 (*n* = 85)	52.94 (45/85)
Adrenal insufficiency or crisis	82 (*n* = 145)	56.55 (82/145)
Hearing disorders	6	3.22 (6/186)
Anal atresia	3	1.61 (3/186)
Maternal virilization	37 (*n* = 96)	38.54 (37/96)

The literature search included publications available up to January 2026. Search terms contained free combinations of PORD, P450 Oxidoreductase Deficiency, Antley-Bixler syndrome, *POR* gene, 46,XY DSD and disorders of sex development. Databases included CNKI, Chinese Medical Journals Network, PubMed, Web of Science and Google Scholar.

Inclusion criteria: ① Cases carrying confirmed pathogenic POR variants; ② Original studies, case reports and case series; ③ Articles providing complete variant information and clinical phenotypic data.

Exclusion criteria: ① Duplicated published datasets; ② Publications with incomplete data that cannot support effective clinical analysis. All citations were imported into EndNote 2025 for automatic deduplication, followed by manual screening to remove repeated records.

^*^The case statistics have already excluded duplicate cases, and only those cases with a definitively detected *POR* gene mutation have been included in the tally.

^#^ 4 patients lacked gender specification and chromosomal karyotyping.

^†^ Only for females with a karyotype of 46,XX.

Beyond the classical endocrine and genital manifestations, this patient exhibited several uncommon abnormalities. Anal atresia (3/186, 1.6%) (19–21) and hearing abnormalities (6/186, 3.2%) (9, 22, 23)are extremely rare reported in PORD. Although the underlying biological mechanisms remain poorly elucidated, severe POR dysfunction during early embryogenesis may indirectly influence cholesterol-dependent signaling pathways, which are involved in processes such as anorectal development and inner ear maturation. These observations suggest that severe biallelic POR defects may be associated with a broader range of developmental processes than previously recognized. In addition, the patient presented with a patent foramen ovale, moderate tricuspid regurgitation, and mild right renal pelvic separation. According to our literature review, these findings are non-characteristic phenotypes of PORD and may represent incidental congenital developmental variations.

This case provides several important clinical implications. First, elevated 17-OHP identified through newborn screening should not be interpreted solely as classical 21-hydroxylase deficiency. When accompanied by skeletal abnormalities or undervirilized 46,XY DSD, PORD should be considered (24). Second, comprehensive genetic analysis, including careful segregation studies to determine allelic configuration and functional assays like RNA-seq for splicing variants, is essential for accurate diagnosis and variant classification (25–27). The reclassification of two variants from VUS to likely pathogenic in this case had direct implications for individual diagnosis, familial carrier screening, and prenatal genetic counseling (28). Finally, patients with PORD require multidisciplinary management to address the complex endocrine, skeletal, auditory, and developmental complications (29). Early recognition and timely intervention are therefore essential for improving clinical outcomes. In this case, timely postnatal evaluation by abnormal newborn screening enabled early clinical intervention and continuous monitoring, effectively preventing life-threatening adrenal crisis and optimizing long-term developmental prognosis (30). During follow-up, the patient was monitored until 14 months of age, with normal growth and developmental milestones observed.

Importantly, this study highlights the clinical value of RNA-based functional assays for variant interpretation and clinical genetic practice. Compared with *in-silico* computational prediction, RNA-level functional testing dynamically reflects the actual biological effect of variants in patient-derived samples, significantly improving the accuracy of variant interpretation (31, 32). Incorporating RNA-based functional assays into diagnostic workflows may improve molecular diagnosis and genetic counseling for rare endocrine disorders with rare variants, especially newly identified splice variants.

### 3.1 Prenatal diagnosis and genetic counseling implications

This case highlights the challenges associated with prenatal diagnosis of PORD and the interpretation of uncertain genetic findings during pregnancy. During the second trimester, maternal serological screening indicated a high risk of trisomy 18; however, subsequent amniotic fluid karyotype analysis and chromosomal microarray analysis revealed no chromosome abnormalities. Retrospective review of the original laboratory data revealed a fetal karyotype of 46,XY, which was subsequently confirmed by postnatal peripheral blood karyotyping. At 23^+^ weeks of gestation, ultrasonography showed fetal left femoral curvature and abnormal foot posture. Amniotic fluid whole-exome sequencing identified three POR variants, including c.1187_1195dup, c.1447G>A, and c.1806 + 4_1806 + 28del. However, all variants were initially classified as VUS, and a definitive prenatal diagnosis could not be established due to insufficient fetal phenotypic evidence at that time. After birth, the infant was confirmed to have 46,XY DSD with discordant genetic and phenotypic sex. The diagnosis of PORD was subsequently established based on the integration of postnatal clinical manifestations, endocrine evaluation, and genetic findings. Taken together, these findings highlight two major challenges in prenatal evaluation of rare genetic disorders: the inherent difficulty of novel or VUS interpretation during pregnancy and the limitations of prenatal phenotyping.

These findings have important implications for prenatal genetic counseling. For fetuses presenting with skeletal abnormalities and VUS identified through prenatal sequencing, clinicians should maintain clinical suspicion and integrate multidimensional evidence, including phenotypic findings, genetic segregation, and functional validation, to improve variant interpretation and facilitate accurate prenatal diagnosis and counseling. Furthermore, our RNA-seq-based splicing analysis in this patient highlights the potential value of transcript-level functional assays for resolving uncertain variants and may serve as a complementary approach to support prenatal genetic diagnosis in selected cases in future clinical practice (33).

### 3.2 Limitations

This study still has several limitations. First, this research adopts a single-case design, which limits the generalizability of our conclusions. All analyses in the present research were based on bioinformatic prediction, and RNA sequencing only reflects gene expression at the transcriptional level. Furthermore, there is currently insufficient evidence to upgrade the pathogenic classification of the c.1447G>A (p.Gly483Ser) variant. Functional verification of the three variants, including functional enzymatic assays, *in-vitro* cellular assays, and *in-vivo* animal model experiments, is required to clarify their pathogenic mechanisms, and further investigations are warranted to address these issues.

## 4 Conclusion

In conclusion, we report a neonate with PORD caused by three novel POR variants and provide integrated clinical, genetic, structural, and transcriptomic evidence supporting variant interpretation. RNA-seq successfully identified aberrant splicing and contributed to the reclassification of a previously uncertain variant. This study expands the genetic and phenotypic spectrum of PORD by describing rare manifestations, including anal atresia and hearing loss, and emphasizes the importance of combining genetic phasing, functional assays, and comprehensive phenotyping for accurate diagnosis and counseling in rare endocrine disorders.

## Data Availability

The original contributions presented in the study are included in the article/**Supplementary Material**. Further inquiries can be directed to the corresponding authors.
