# Supplementary material for: Novel compound heterozygous POR variants in a neonate with Antley-Bixler syndrome and 46,XY DSD: a case report and literature review
Source: Front Endocrinol (Lausanne). 2026 Aug 28;17:1896956. doi: 10.3389/fendo.2026.1896956 (PMC13561933; doi:10.3389/fendo.2026.1896956)
Supplement: Supplementary file 1 [file SupplementaryFile1.docx]

**Supplementary Figure 1**

***POR* gene variants are predominantly missense mutations.**

**Supplementary Figure 2**

**Among the missense mutations, p.R457H is the most frequently detected variant.**
